# The presence and outcome of biliary sphincter disorders in liver-transplant recipients according to the Rome IV classification

**DOI:** 10.1093/gastro/goab025

**Published:** 2021-06-28

**Authors:** Alejandro Fernandez-Simon, Oriol Sendino, Karina Chavez-Rivera, Henry Córdova, Jordi Colmenero, Gonzalo Crespo, Yilliam Fundora, Franco Samaniego, Pablo Ruiz, Constantino Fondevila, Miquel Navasa, Andrés Cárdenas

**Affiliations:** 1 GI Unit, Institut Clínic de Malalties Digestives i Metabòliques, Hospital Clínic, University of Barcelona, Barcelona, Spain; 2 Institut d’Investigacions Biomèdiques August Pi-Sunyer (IDIBAPS) and Ciber de Enfermedades Hepáticas y Digestivas (CIBEREHD), Barcelona, Spain; 3 Liver Transplant Unit, Institut Clínic de Malalties Digestives i Metabòliques, Hospital Clínic, University of Barcelona, Barcelona, Spain

**Keywords:** sphincter of Oddi dysfunction, liver transplantation, biliary sphincterotomy, papillary stenosis, functional biliary sphincter disorder

## Abstract

**Background:**

Biliary sphincter disorders after liver transplantation (LT) are poorly described. We aim to describe the presence and outcome of patients with papillary stenosis (PS) and functional biliary sphincter disorders (FBSDs) after LT according to the updated Rome IV criteria.

**Methods:**

We reviewed all endoscopic retrograde cholangiopancreatographies (ERCPs) performed in LT recipients between January 2003 and December 2019. Information on clinical and endoscopic findings was obtained from electronic health records and endoscopy databases. Laboratory and clinical findings were collected at the time of ERCP and 1 month after ERCP.

**Results:**

Among the 1,307 LT recipients, 336 underwent 849 ERCPs. Thirteen (1.0%) patients met the updated Rome IV criteria for PS [former sphincter of Oddi dysfunction (SOD) type I] and 14 patients (1.0%) met the Rome IV criteria for FBSD (former SOD type II). Biliary sphincterotomy was performed in 13 PS and 10 FBSD cases. One month after sphincterotomy, bilirubin, gamma-glutamyl transferase and alkaline phosphatase levels decreased in 85%, 61%, and 92% of those in the PS group (*P* = 0.019, 0.087, and 0.003, respectively) and in 50%, 70%, and 80% of those in the FBSD group (*P* = 0.721, 0.013, and 0.093, respectively). All the 14 patients initially suspected of having a FBSD turned out to have a different diagnosis during the follow-up.

**Conclusions:**

PS after LT is uncommon and occurs in only 1% of LT recipients. Our data do not support the presence of an FBSD after LT. Sphincterotomy is a safe and effective procedure in LT recipients with PS.

## Introduction

Biliary sphincter dysfunction is a clinical syndrome that occurs in patients with biliary-type pain due to a functional abnormality of the sphincter of Oddi [1]. Due to the lack of a standardized system to diagnose this entity, it is difficult to estimate the real prevalence in the general population. It is generally accepted that it is more frequently recognized after cholecystectomy [2], with a reported incidence of <1% in large consecutive series of patients after cholecystectomy and ≤14% of selected patients complaining of post-cholecystectomy symptoms [3, 4]. A possible explanation for this alteration is the suppression of the normal inhibitory effect of cholecystokinin due to the denervation of nerve fibers between the cystic duct and the sphincter of Oddi, leading to a hypertonic sphincter [5–7]. This simple explanation for the phenomenon that seems to cause biliary obstruction and pain was recently challenged, especially in those patients with biliary pain without significant abnormalities in imaging or laboratory studies [8], suggesting that the pathophysiology of biliary sphincter dysfunction is more complex. Nociceptive sensitization may also play a key role as the cause of pain. Whether biliary sphincter hypertension is relevant as a cause or as a marker of this functional disorder remains unclear [9, 10]. The traditional classification of sphincter of Oddi dysfunction (SOD) included three types according to the presence or absence of biliary pain plus elevation of liver enzymes and/or presence of dilated biliary ducts. SOD type I was defined as the presence of all three conditions; type II patients presented with biliary pain and elevated liver enzymes or dilated bile ducts; and type III was considered when biliary pain alone was present. In the most recent Rome IV consensus, this classification was considered outdated, as most type I patients present a papillary stenosis (PS) rather than a functional disorder and have an excellent response after sphincterotomy [10]; type III patients have no response to sphincterotomy [8, 11]; finally type II has now been renamed as suspected functional biliary sphincter disorder (FBSD) [10].

The pathogenesis of biliary sphincter disorder in liver transplantation (LT) recipients is poorly understood [12]. More factors need to be taken into account in the post-LT setting, such as the use of a T-tube (when its distal end protrudes through the papilla), the presence of opportunistic infections like cytomegalovirus (CMV), and post-surgical edema [13, 14]. The diagnosis of FBSD in this context is more challenging than in the general population perhaps because some LT recipients may not present with typical abdominal pain due to hepatic denervation after the surgery and immunosuppression [12, 15]. Therefore, suspicion of a biliary sphincter disorder in LT recipients can be considered when cholestasis and/or dilation of bile ducts appear in the absence of bile stones or other structural abnormalities [16]. Confirmation of biliary sphincter hypertension requires sphincter manometry—a technique that is complex, lacks reproducibility of results, and has significant adverse effects; therefore, it is not routinely performed in LT recipients [17]. Several reports indicate that there is a high response with the normalization of liver in enzymes after sphincterotomy LT recipients with suspected SOD, so this has been generally accepted as a pragmatic way to confirm diagnosis [18].

Based on traditional criteria, the incidence of SOD ranges between 2% and 7% in post-LT recipients [19–23], reaching ≤16% in one large series in which the response to sphincterotomy was not evaluated [24]. However, the updated Rome IV consensus for biliary sphincter disorders did not consider the diagnostic criteria in patients after LT [10]. In most studies, patients with suspected SOD had dilation of bile ducts and elevation of liver enzymes, which suggest PS rather than FBSD. Furthermore, given that the absence of biliary dilation and exclusion of any biliary structural abnormalities (stones, sludge, strictures, etc.) are required to establish FBSD in LT recipients, confirmation of this diagnostic suspicion is especially challenging if other conditions such as graft rejection, hepatitis C virus (HCV) recurrence, or other intrahepatic causes of cholestasis are not excluded first. Most of the reports lack a follow-up, which is necessary in order to rule out other causes of cholestasis after LT. Thus, the primary objective was to describe the presence of biliary sphincter disorders in a large cohort of LT recipients based on the Rome IV consensus and their outcome after endoscopic retrograde cholangiopancreatography (ERCP) and biliary sphincterotomy.

## Patients and methods

### Study design and study subjects

This is a retrospective analysis of a prospectively collected database of all patients who underwent ERCP after LT. This analysis was conducted in a tertiary care hospital where 80–100 liver transplants are performed on a yearly basis and >450 ERCPs are performed annually. We performed an analysis of all ERCPs performed in LT recipients with duct-to-duct anastomosis in our institution between 1 January 2003 and 31 December 2019, inclusive. Data were collected and entered after each case. The electronic medical records and endoscopy database of our hospital were accessed to abstract demographic, clinical, and endoscopic data. The study protocol conforms to the ethical guidelines of the 1975 Declaration of Helsinki as reflected in prior approval by the institutions’ human ethic review board (approval No. 20128003).

Patients with abnormal liver enzymes in whom no cause of rejection or recurrence of the disease was suspected with abnormal ultrasound or abnormal cross-sectional imaging that raised suspicion of biliary complications after LT were referred to ERCP. Biliary dilation was defined if the common bile duct (CBD) of the implant was dilated by >7 mm. In addition, an image consistent with an anastomotic stricture was defined as a short narrowing of the bile of <5 mm at the site of the anastomosis. We excluded all patients with CBD stones.

### ERCP procedure

All procedures were performed with monitored sedation under the supervision of an anesthesiologist with levels from moderate to general anesthesia and in the prone position. All patients received antibiotic prophylaxis before ERCP. Using a therapeutic duodenoscope, the bile duct was cannulated using a wire-guided sphincterotome. If cannulation failed, biliary access was achieved by advanced techniques that included using a double-wire technique or precut sphincterotomy (freehand or over a pancreatic duct stent). Once deep cannulation was achieved, a cholangiography was performed. Biliary sphincterotomy was performed using the standard technique.

### Outcome measurement

Given that all patients presented with cholestasis, we established two groups after ERCP according to the Rome IV criteria for FBSD: (i) patients with dilation of bile ducts confirmed at ERCP were defined as PS (former SOD I); and (ii) patients with normal bile ducts were defined as FBSD (former SOD II). Laboratory results and clinical data were collected at the time of ERCP and 1 month after ERCP. Definitions of individual adverse events and their severity after ERCP (i.e. pancreatitis, cholangitis, hemorrhage, perforation) were defined by criteria as established by Cotton *et al.* [25, 26]. Mild events were considered when hospitalization was prolonged by 2–3 days, moderate by 4–10 days, and severe by >10 days.

### Statistical analysis

Categorical variables were expressed as numbers and percentages. Since the sample size of those that met the criteria for PS and FBSD was small, continuous variables were expressed in medians and interquartile ranges (IQRs). Differences in laboratory parameters before and after ERCP were studied using a Wilcoxon signed-rank test. *P*-values of <0.05 were considered statistically significant. The software used was the SPSS Statistical Package (version 23.0, SPSS Inc).

## Results

**Figure 1.**
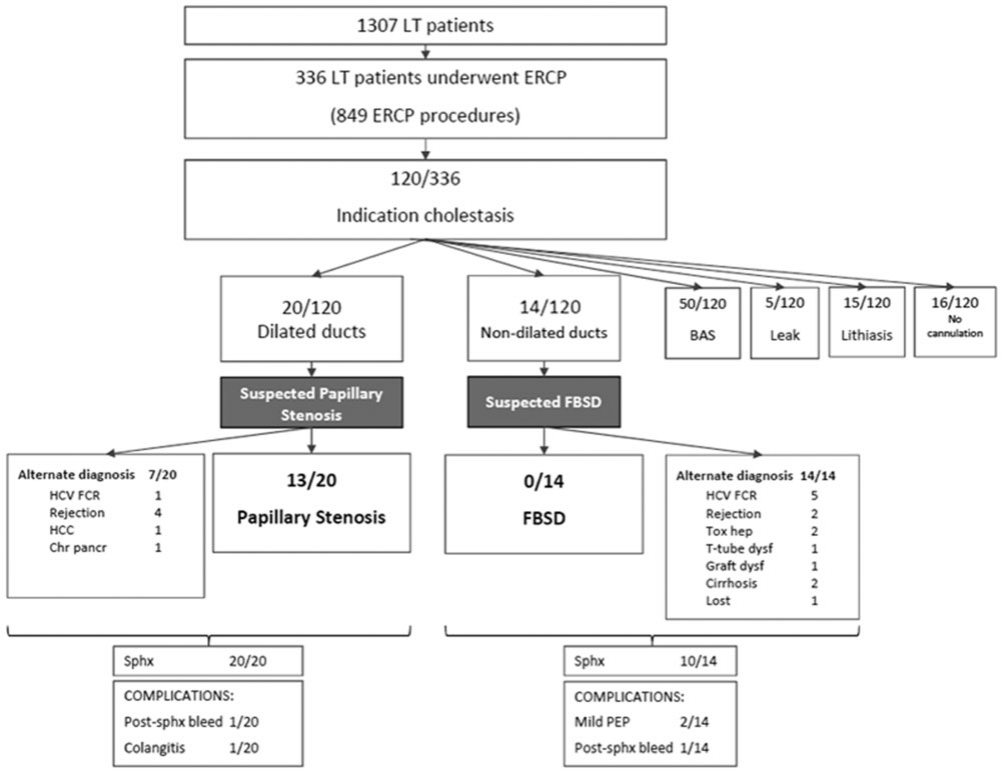
Patient flowchart LT, liver transplantation; ERCP, endoscopic retrograde cholangiopancreatography; BAS, biliary anastomosis stricture; HCV, hepatitis C virus; FCR, fibrosing cholestatic recurrence; HCC, hepatocellular carcinoma; Chr pancr, chronic pancreatitis; sphx, spincterotomy; post-sphx bleed, post-sphincterotomy bleeding; FBSD, functional biliary sphincter disorder; Tox hep, toxic hepatitis; dysf, dysfunction; PEP, post-ERCP pancreatitis.

A total of 1,307 patients underwent LT from 1 January 2003 and 31 December 2019 at Hospital Clinic in Barcelona, among whom 336 patients underwent 849 ERCPs for different indications such as strictures, bile leaks, CBD stones, and/or cholestasis. Persistent cholestasis with or without dilation of bile ducts was the indication for ERCP in 120 patients (36%). None of these patients presented with abdominal pain. In 20 patients, ERCP showed dilated bile ducts without any filling defects or strictures. In these, 13 met the Rome IV criteria for PS (1%, 13/1,307); in 7, an alternate cause of cholestasis was identified. Fourteen had a normal cholangiography and these patients met the Rome IV criteria for FBSD (1%, 14/1,307). In the remaining patients, 50 had a biliary anastomotic stricture that was not detected in previous imaging tests, 5 had a bile leak, 15 had CBD stones or sludge, and no cannulation was performed in 16 cases (Figure 1). All patients with PS (*n* = 13) and suspected FBSD (*n* = 14) had a native papilla.

Demographic data and initial laboratory parameters of the patients who met criteria for PS and FBSD are shown in Table 1. The main indications for LT were viral hepatitis and alcoholic liver disease. All patients were Spanish Caucasians and had a duct-to-duct anastomosis. All the 13 patients with PS and 71% (10/14) of patients with FBSD underwent biliary sphincterotomy. Only four patients in the FBSD group did not undergo sphincterotomy due to acute rejection (*n* = 1), recurrent HCV (*n* = 1), graft dysfunction (*n* = 1), and toxic hepatitis (*n* = 1). All patients were followed up for ≥1 month. All patients initially suspected of having a FBSD had a different diagnosis during the follow-up after the ERCP (Figure 1). Time to final diagnosis in FBSD group from the index ERCP was <30 days in all patients.

**Table 1.** Demographics and initial laboratory parameters in patients who met the criteria for papillary stenosis (PS) and functional biliary sphincter disorder (FBSD) before endoscopic retrograde cholangiopancreatography (ERCP)

Characteristic	PS (*n* = 20)	FBSD (*n* = 14)
Male, *n* (%)	11 (55)	7 (50)
Age, median (IQR)	55.8 (12.3)	57.1 (10.6)
Etiology, *n* (%)		
Hepatitis C virus	12 (60)	9 (64)
Hepatitis B virus	2 (10)	0 (0)
Alcohol	3 (15)	2 (14)
Fulminant hepatic failure	2 (10)	3 (21)
Primary biliary cirrhosis	1 (4.3)	0 (0)
Laboratory parameters, median (IQR)		
White cell blood count, 10^9^/L	6,000 (4,425)	4,680 (6,720)
Hemoglobin, g/L	10.8 (4.3)	10.4 (1.6)
Platelets, 10^9^/L	176 (132)	77 (176)
Alanine transaminase, U/L	85 (123)	85 (95)
Aspartate aminotransferase, U/L	57 (52)	88 (71)
Total bilirubin, mg/dL	2.4 (3.0)	4.4 (13.7)
Alkaline phosphatase, U/L	767 (1088)	647 (767)
γ-glutamyl transferase, U/L	425 (465)	536 (679)
International normalized rate	1.1 (0.2)	1.1 (0.3)
Creatinine, mg/dL	1.2 (0.6)	1.1 (0.3)

**Figure 2.**
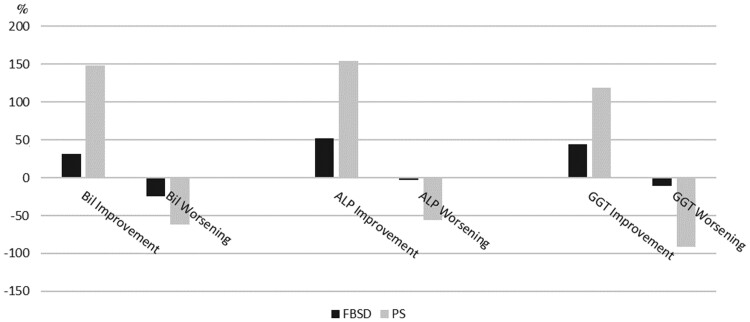
Laboratory-test evolution (proportion) in the PS group and FBSD group before and after endoscopic retrograde cholangiopancreatography FBSD, functional biliary sphincter disorder; PS, papillary stenosis; Bil, bilirubin; ALP, alkaline phosphatase; GGT, gamma-glutamyl transferase.

In order to evaluate the effect of sphincterotomy in both groups, laboratory data were analysed at the time of sphincterotomy and 1 month later. In the PS group, total bilirubin and alkaline phosphatase (ALP) significantly decreased after sphincterotomy in 85% (11/13) and 92% (12/13), respectively; γ-glutamyl transferase (GGT) decreased in 62% (8/13), although not reaching statistical significance. In the FBSD group, total bilirubin, GGT, and ALP decreased after sphincterotomy in 50% (5/10), 70% (7/10), and 86% (8/10) of the patients (Table 2). Twelve out of 14 patients in the FBSD group had a liver biopsy performed after the ERCP: 5 had HCV fibrosing cholestatic recurrence, 2 acute rejections, 2 toxic hepatitis, 2 “*de novo*” cirrhosis, and 1 had graft dysfunction. The evolution of the bilirubin, GGT, and ALP levels before and after sphincterotomy in those with PS is shown in Figure 2.

**Table 2.** Laboratory findings before and after endoscopic retrograde cholangiopancreatography (ERCP) in patients with papillary stenosis (PS) and functional biliary sphincter disorder (FBSD)

Laboratory parameter, median (IQR)		Before ERCP	After ERCP	*P*-value
PS (*n* = 13)	TBil, mg/dL	2.3 (1.8)	0.9 (0.9)	0.019
	GGT, U/L	338 (423)	130 (454)	0.087
	ALP, U/L	697 (801)	222 (278)	0.003
Suspected PS with alternative diagnosis (*n* = 7)	TBil, mg/dL	3.6 (13.0)	2.2 (4.8)	1.000
	GGT, U/L	716 (659)	1933 (2,215)	0.398
	ALP, U/L	1,458 (1611)	1,277 (2,081)	0.735
FBSD with sphincterotomy (*n* = 10)	TBil, mg/dL	1.95 (9.7)	2.2 (5.6)	0.721
	GGT, U/L	487 (512)	282 (583)	0.013
	ALP, U/L	598 (832)	256 (385)	0.093
FBSD no sphincterotomy (*n* = 4)	TBil, mg/dL	15 (27.5)	0.9 (22.4)	0.465
	GGT, U/L	1,143 (1,381)	168 (356)	0.068
	ALP, U/L	872 (1,411)	201.5 (1,006)	0.068

TBil, total bilirubin; GGT, gamma-glutamyltransferase; ALP, alkaline phosphatase.

Complications after ERCP in the PS group included post-sphincterotomy bleed (*n* = 1) that required a prolonged hospital stay and severe cholangitis (*n* = 1). In the FBSD group, two patients presented mild post-ERCP pancreatitis and had one post-sphincterotomy bleeding.

## Discussion

There is a broad range of biliary complications that can arise after LT. The reported incidence of biliary complications after LT ranges between 15% and 25%, and these complications include biliary strictures, anastomotic leaks, choledocholithiasis, and biliary casts that can occur after any type of LT [18, 20, 21, 27]. Among all these problems, biliary sphincter disorder is the less frequent biliary complication after LT, as reported elsewhere [16, 18, 28]. The pathophysiology is not fully understood and its incidence is difficult to evaluate given that, for a definite diagnosis, it requires thorough exclusion criteria that can rarely be met in these patients. To date, there are no specific studies that have evaluated this entity in LT recipients. Our study provides the largest cohort of LT recipients to date in which this uncommon complication is specifically analysed.

After the consensus conference of gallbladder and sphincter of Oddi disorders (Rome IV [10]), criteria to establish SOD changed and, as such, these considerations should be taken into account in LT recipients. To our knowledge, this is the first study to evaluate biliary sphincter disorders in LT recipients. The data in this analysis of a large cohort of LT recipients indicate that none met the criteria for an FBSD. All patients who presented with a high suspicion of FBSD at ERCP were then shortly diagnosed in follow-up with other common post-LT complications such as fibrosing cholestatic HCV recurrence or graft rejection, which ruled out FBSD. As a result, these patients did not show a significant improvement of liver enzymes after sphincterotomy. These data suggest that a diagnosis of FBSD is difficult to establish and likely not present after LT.

Of note, 7 out of 20 patients who had a high pre-diagnostic likelihood of PS did not improve after sphincterotomy. This response rate can be explained by the presence of other post-LT complications. When these patients were excluded from the analysis, response to sphincterotomy improved significantly. This indicates that suspected PS in the setting of LT should not preclude investigation for other causes of cholestasis. The analysis and follow-up of cases suspected of having a biliary sphincter disorder highlight the difficulty in securing a diagnosis of exclusion at the time of ERCP. There is significant overlap of alternate diagnoses that can arise in the post-ERCP follow-up period. These considerations need to be taken into account when addressing biliary obstruction in such patients given that the lack of strictures of biliary defects (stones or casts) on imaging does not imply that these patients (cholestasis and dilated bile ducts) have PS and to a lesser extent FBSD. Although performing sphincterotomy in such patients could be considered a therapeutic/diagnostic trial, strict follow-up is necessary because an additional diagnosis may appear. The results of this study and our experience indicate that PS is a real entity and that FBSD is probably not present in LT recipients. Our findings indicate that before considering an ERCP with sphincterotomy, a careful assessment of other intrahepatic causes of cholestasis need to be ruled out before performing this procedure.

The results of this study indicate that the response to sphincterotomy should be considered as a condition to establish the definite diagnosis of PS in LT recipients, but at least a 4-week follow-up period is required in order to secure this diagnosis. Although denervation after LT leads to an increase in basal sphincter of Oddi pressure and increased pressure in the CBD [29], there few studies that have directly assessed sphincter of Oddi pressures in post-LT patients. Our results indicate that the response to sphincterotomy in LT patients with true PS is excellent, as reported by other groups, and sphincter of Oddi manometry is probably not necessary in these cases [18]. In the general population, the response to sphincterotomy depends on the type of alteration, with response rates of 90%, 75%, and 50% for former SOD biliary types I, II, and III, respectively [3, 30]. Sphincter of Oddi manometry may play a role in the study of FBSD (former type II SOD) but recent data indicate that both manometry and biliary sphincterotomy are not justified in former type III SOD [8, 10, 11]. In LT recipients, on the other hand, data are scarce and indicate that the response to sphincterotomy is high when patients show persistent and uniform dilation of bile ducts, which would indicate PS rather than dyskinesia [18, 21, 31]. To date, there are no studies assessing the incidence of FBSD in LT recipients. In our experience, sphincterotomy had a poor outcome in LT recipients with an initial suspicion of FBSD and none could have this diagnosis confirmed at the end of the follow-up.

Limitations of our study include its retrospective and single-center nature along with a low number of patients presenting FBSD or PS, which precluded the use of parametric analysis. In addition, these are consecutive cases of patients who were only referred for ERCP, so we could not evaluate patients who had biliary dilation and/or abnormal liver enzymes who did not undergo an ERCP and instead underwent other procedures such as percutaneous transhepatic cholangiography or surgery.

In conclusion, PS is a rare cause of cholestasis in LT recipients. When the diagnosis is confirmed after a follow-up period of 4 weeks, response rates after sphincterotomy are excellent. In this series of LT recipients, FBSD was not present and, in this situation, patients do not benefit from a biliary sphincterotomy. More studies are needed to clarify whether it is feasible to consider FBSD as a real condition in LT recipients.

## Authors’ Contributions

A.F.S. and A.C. conceived of and designed the project. A.F.S., O.S., K.C.R., H.C., J.C., G.C., Y.F., F.S., and P.R. collected the data. A.F.S., A.C., C.F., and M.N. analysed and interpreted the data. A.F.S. and A.C. drafted the manuscript. All authors read and approved the final manuscript.

## Funding

A.C. is funded by the Instituto de Salud Carlos III and Plan Estatal de Investigación Ciéntifica y Técnica y de Innovación [Grant No. PI19/00752] and has received funding for this work by ‘Fundación Marta Balust’.
